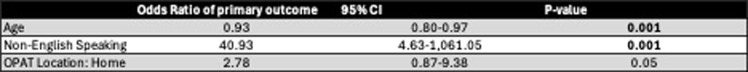# 358 Short-Term HRQoL After Acute Respiratory Illness in Adults Aged ?65 Years: Findings From the U.S. Flu VE Network 2024-2025

**DOI:** 10.1017/ash.2026.10696

**Published:** 2026-06-23

**Authors:** Maureen Belizaire, HeeEun Kang, Nina Clark

**Affiliations:** 1 Loyola University Stritch School of Medicine; 2 Loyola University Medical Center

## Abstract

**Introduction:** Outpatient parenteral antimicrobial therapy (OPAT) allows patients to receive intravenous antibiotics safely outside the hospital and is an important strategy to reduce inpatient hospital stays, healthcare costs, and exposure to nosocomial pathogens. However, OPAT carries risks, including adverse drug events, repeat healthcare encounters, and treatment complications. Prior studies suggest certain populations such as Black/African American patients may be at higher risk for adverse outcomes, underscoring the need to evaluate OPAT program performance. We aimed to characterize clinical outcomes among patients enrolled in the Loyola University Medical Center (LUMC) OPAT program and identify factors associated with adverse events. **Methods:** We conducted a retrospective case-control study of adults enrolled in the LUMC OPAT program between 10/01/24 – 12/31/24. Patients were identified through OPAT flowsheets, a specialized Epic flowsheet created to follow patients enrolled in OPAT. Inclusion criteria were age ?18 years and completion of OPAT at LUMC. Patients who did not complete OPAT at LUMC, lacked documentation, or followed with non-LUMC ID were excluded. Cases were defined as those who suffered an OPAT complication, defined as any of the following: 30-day OPAT or infection related ER visit and hospital admissions, medication adverse events, and vascular access complications. Controls were patients who did not have an OPAT complication. Data collected encompassed patient demographics, comorbidities, and OPAT related clinical characteristics. Univariable and multivariable analysis was conducted using R, statistical significance set to p<0.05. **Results:** Of 97 patients identified, 74 met the inclusion criteria (Figure 1). 27 (36%) experienced an OPAT complication (Table 1). OPAT complications were more common in patients who were younger (median age 52 vs 64 years, p<0.01) and receiving OPAT at home (63% vs 32%, p=0.04). In multivariable analysis, younger age, and non-English speaking status were strongly associated with OPAT complications. **Conclusion:** Younger age and non-English speaking status were significantly associated with OPAT complications. Receiving OPAT at home demonstrated a trend toward increased risk. Race, ethnicity, zip code, insurance status, and underlying comorbidities were not associated with complications. Microorganism and vascular access type did not differ significantly between groups. A larger study is planned to confirm associations identified from this study, and to develop interventions to help improve outcomes for at-risk populations.

**Figure:**
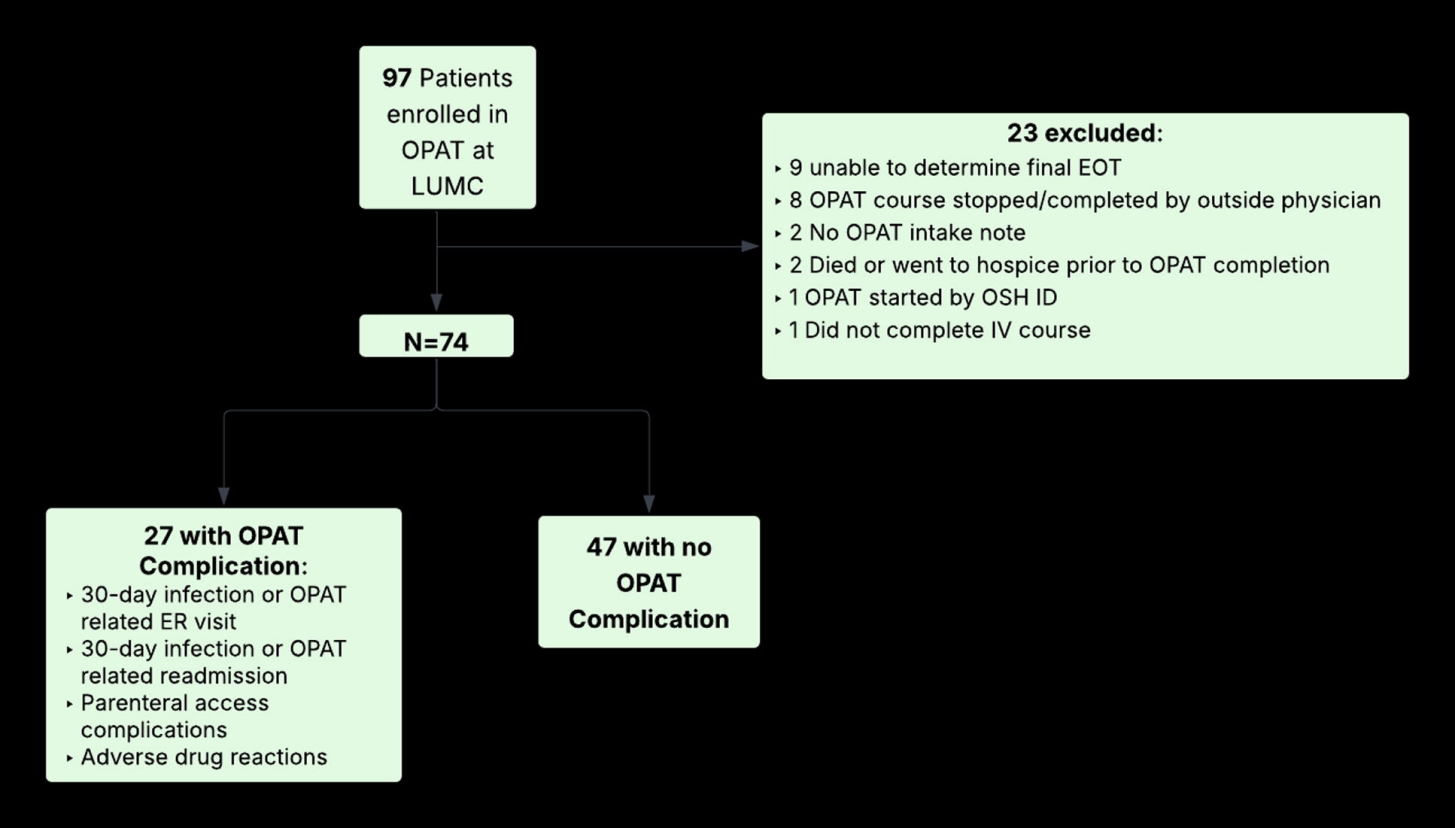

**Figure:**
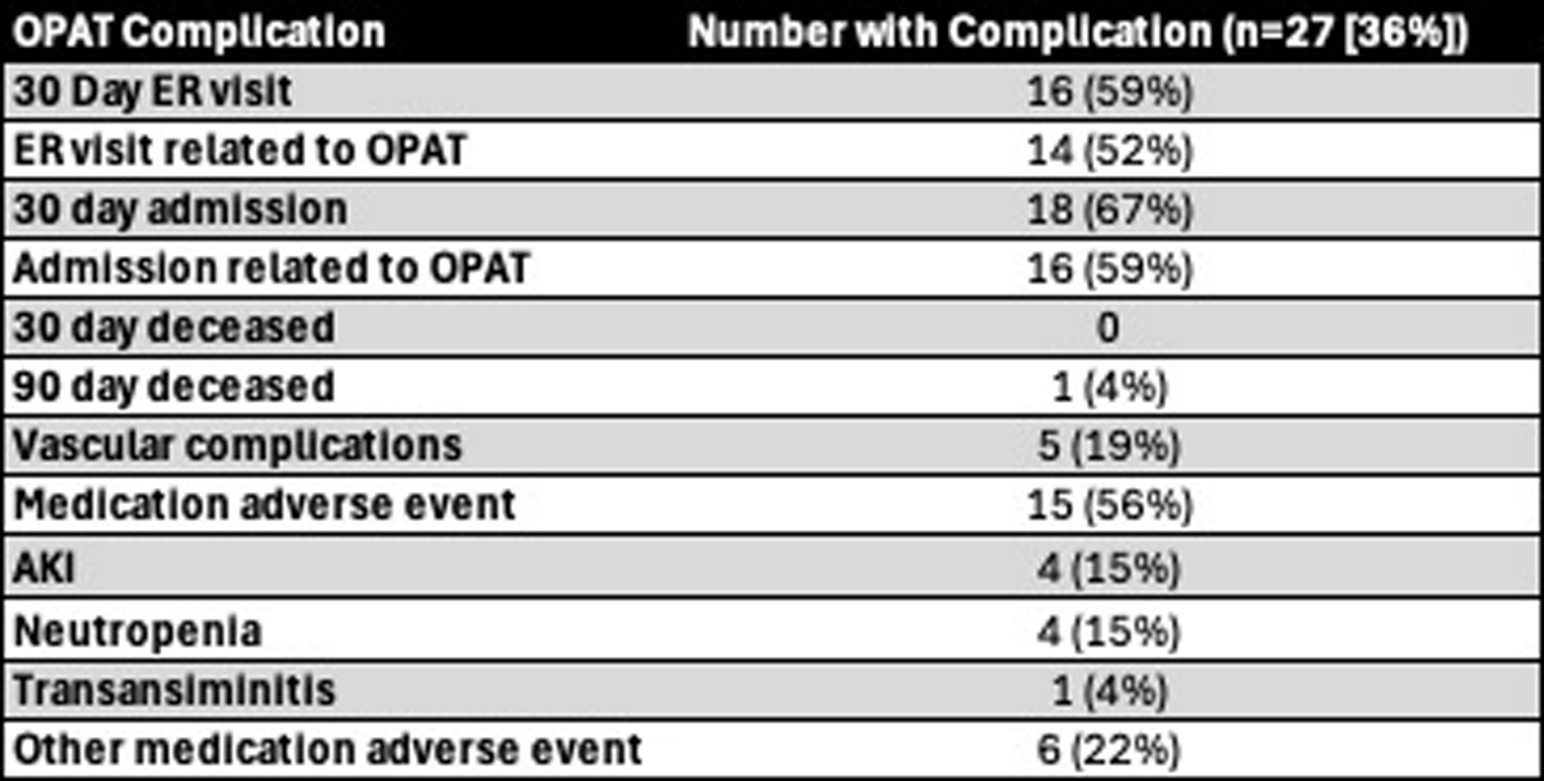

**Figure:**
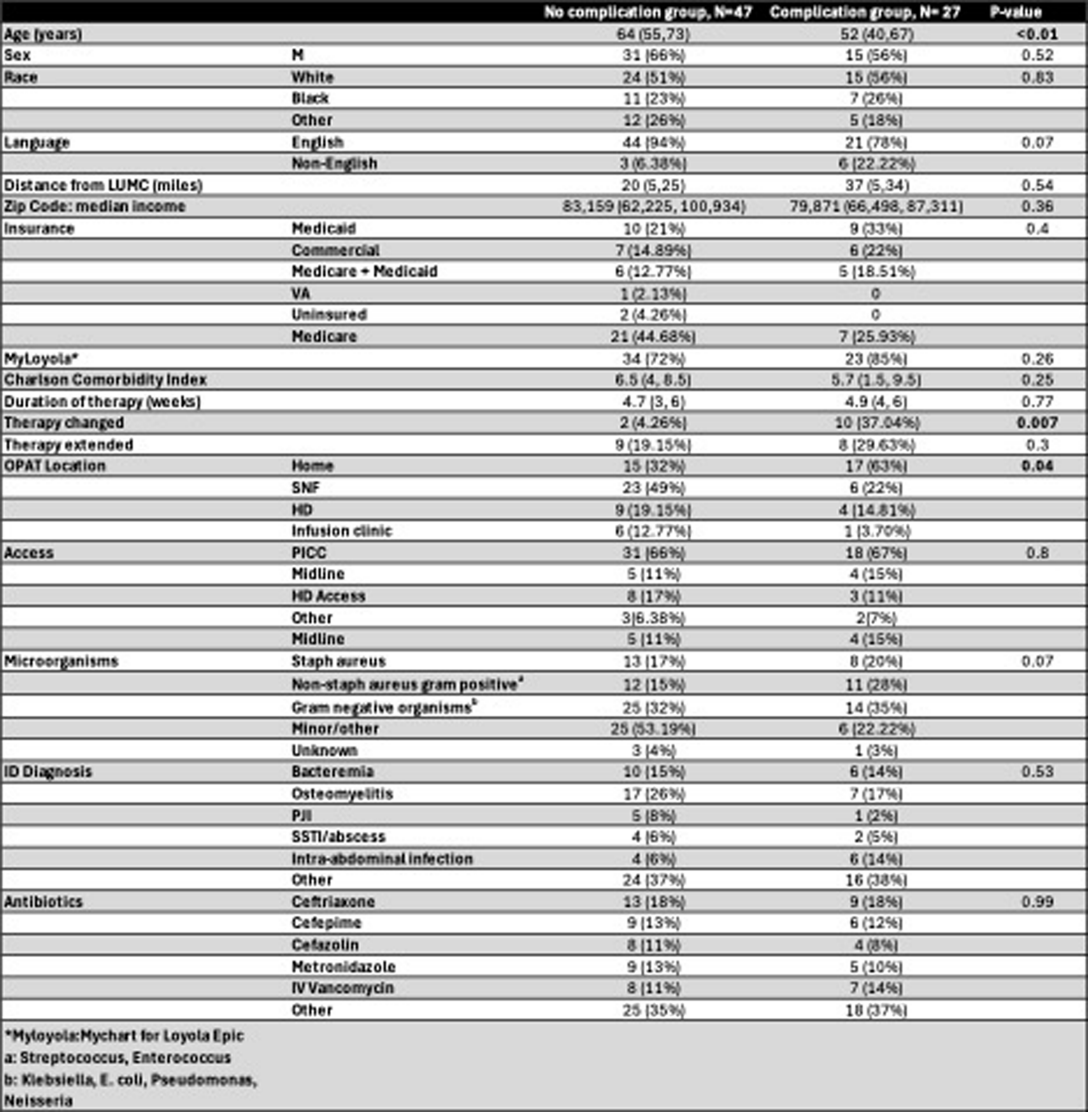

**Figure:**